# Role of endoscopic management in synthetic sling/mesh erosion following previous incontinence surgery: a systematic review from European Association of Urologists Young Academic Urologists (YAU) and Uro-technology (ESUT) groups

**DOI:** 10.1007/s00192-019-04087-5

**Published:** 2019-08-29

**Authors:** Sulaiman Sadaf Karim, Amelia Pietropaolo, Andreas Skolarikos, Omar Aboumarzouk, Panagiotis Kallidonis, Thomas Tailly, Vincent de Coninck, Etienne Xavier Keller, Bhaskar Kumar Somani

**Affiliations:** 1 University Hospital Southampton NHS Trust, Southampton, UK; 2 2nd Department of Urology, Sismanoglio Hospital, National and Kapodistrian University of Athens, Athens, Greece; 3 Department of Urology, Queen Elizabeth University Hospital, Glasgow, UK; 4 Department of Urology, University of Patras, Patras, Greece; 5 University Hospitals Ghent, Ghent, Belgium; 6 Department of Urology, AZ Klina, Brasschaat, Belgium; 7 Department of Urolog, University Hospital Zurich, University of Zurich, Zurich, Switzerland; 8 Department of Urology, University Hospital Southampton NHS Trust, Southampton, SO16 6YD UK

**Keywords:** Endoscopic, Endoscopic removal, Endoscopy, Laser, Mesh, Sling, Tape, Incontinence, TVT, TOT, Mesh erosion

## Abstract

**Introduction and hypothesis:**

Foreign body (FB) erosion is now recognized as a major long-term complication following previous incontinence surgery. The aim of our systematic review was to ascertain the outcomes of endoscopic management in synthetic sling/mesh erosion following previous gynaecological surgery.

**Methods:**

A systematic review in line with PRISMA and Cochrane guidelines was conducted for all English language articles between 1996 and December 2018 for all articles reporting on endoscopic surgical management for eroded FB following previous sling/mesh procedure for incontinence.

**Results:**

Our search produced 931 articles of which 20 articles (198 patients) were included in our review; 149 (75%) had tension-free vaginal tapes (TVT) or tension-free obturator tapes (TOT) as their initial procedure. The site of mesh erosion was the bladder in 134 patients (68%) of which 12 (6%) were in the bladder neck. Urethral mesh erosion was seen in 63 patients (32%) across all studies.

The treatment of eroded mesh was by laser and endoscopic excision using an electrode loop or laparoscopic scissors in 108 (55%) and 90 (45%) patients respectively. The initial/final success rate with laser and endoscopic excision was 67%/92% and 80%/98% respectively. The overall complication rates were 24% and 28% in laser and endoscopic groups respectively of which 21% in each group were stress urinary incontinence.

**Conclusions:**

Endoscopic management of FB erosion is an effective minimally invasive technique with good outcomes and minimal morbidity. Management with the use of holmium laser is gaining momentum and could be attempted before open surgical removal. There is a need for comparative data between open surgical excision and endoscopic excision to help better describe the patient’s most likely to benefit from the endoscopic technique.

## Introduction

Over the last 2 decades the treatment of stress urinary incontinence (SUI) has shifted to a midurethral sling (MUS) or a mesh-based bladder neck procedure [1]. Although the surgery was believed to be relatively safe, there has been a steep rise in the number of reported cases of their erosion into the lower urinary tract [2–10]. Mesh erosion was first reported in 2001 but is now recognized as a major long-term complication of MUS surgery with an incidence rate of between 0.6 and 5.4% [2, 11].

Although the true extent of mesh erosion seems to be under-reported, due to an alarming rise of reported cases in 2018, there was a temporary ban on all MUS procedures in the UK [12]. These erosions and related complications seem to have happened a few years after the initial surgery, with patients presenting with recurrent urinary tract infections (rUTIs), lower urinary tract symptoms (LUTS), recurrent SUI, haematuria and bladder or pelvic pain [13–15]. While open surgical excision via vaginal or abdominal route could be performed with urethrotomy and reconstruction in some cases [16], increasingly there are papers that suggest that they can be managed endoscopically.

Endoscopic management of eroded foreign body (FB) can be performed by intravesical resection of the mesh with electrode loop via a resectoscope or laparoscopic scissors [17–20], although it has been increasingly popular to use transurethral endoscopic resection using holmium laser [21]. These are minimally invasive approaches to a complex clinical problem.

The aim of our systematic review was to ascertain the outcomes of endoscopic management in synthetic sling/mesh erosion following previous incontinence surgery.

## Methods

### Evidence acquisition

- *Inclusion criteria:*

1. Endoscopic surgical interventions for FB excision including laser procedures for adult patients who previously underwent synthetic sling/mesh procedure for incontinence.
2. Studies in English language reporting on a minimum of three patients.

- *Exclusion criteria:*

1. Animal studies, case reports and laboratory studies
2. Open surgical interventions
3. Male sling procedures

### Search strategy and study selection

A systematic literature search was conducted according to the Cochrane review and Preferred Reporting Items for Systematic Reviews and Meta-Analyses (PRISMA) protocol [22, 23]. Medical subject headings (MeSH) terms used, but not limited to, were: ‘endoscopic’, ‘laser’, ‘holmium’, ‘mesh’, ‘erosion’, ‘sling’, ‘TVT’, ‘tension-free vaginal tape’, ‘transobturator tape’, ‘TOT’, ‘urethral’, ‘transurethral’, ‘resection’, ‘removal’, ‘incontinence’, ‘surgical’, ‘incision’ and ‘intervention’. Boolean operators (AND, OR) were used to refine the search. The search strategy was conducted to find relevant articles from CINAHL (1996–December 2018), EMBASE (1996–December 2018), Ovid Medline (1996–December 2018), Cochrane Library (2018), Scopus (1996–December 2018), Clinicaltrials.gov, Google Scholar and individual urological journals. The search was confined to all English language articles between January 1996 and December 2018. All original studies were included and two reviewers (S.S and B.K.S.) identified all studies independently; discrepancies were resolved by mutual consensus. Where additional information or clarification was needed, the primary authors of the studies were contacted directly.

The PICO statement for this review is as follows: the population examined was adults who underwent synthetic sling procedures for incontinence. There was no comparative group, and the outcomes were to ascertain the results of endoscopic management following sling erosion.

### Data extraction and analysis

The following variables were extracted from each study: journal and year of publication, study type, time period, presenting symptom, procedure type, time to presentation following initial surgery, location of mesh erosion, complications and their management, success rate and follow-up. We used the standard IUGA/ICS terminology for reporting mesh-associated complications in this manuscript [24]. Data were collated using Microsoft Excel (Microsoft Corp., Redmond, WA, USA), version 12.2.4.). There was a substantial degree of heterogeneity between studies in both design and reporting, and hence our analysis was limited to narrative synthesis and pooled analysis of mean results.

## Results

**Fig. 1:**
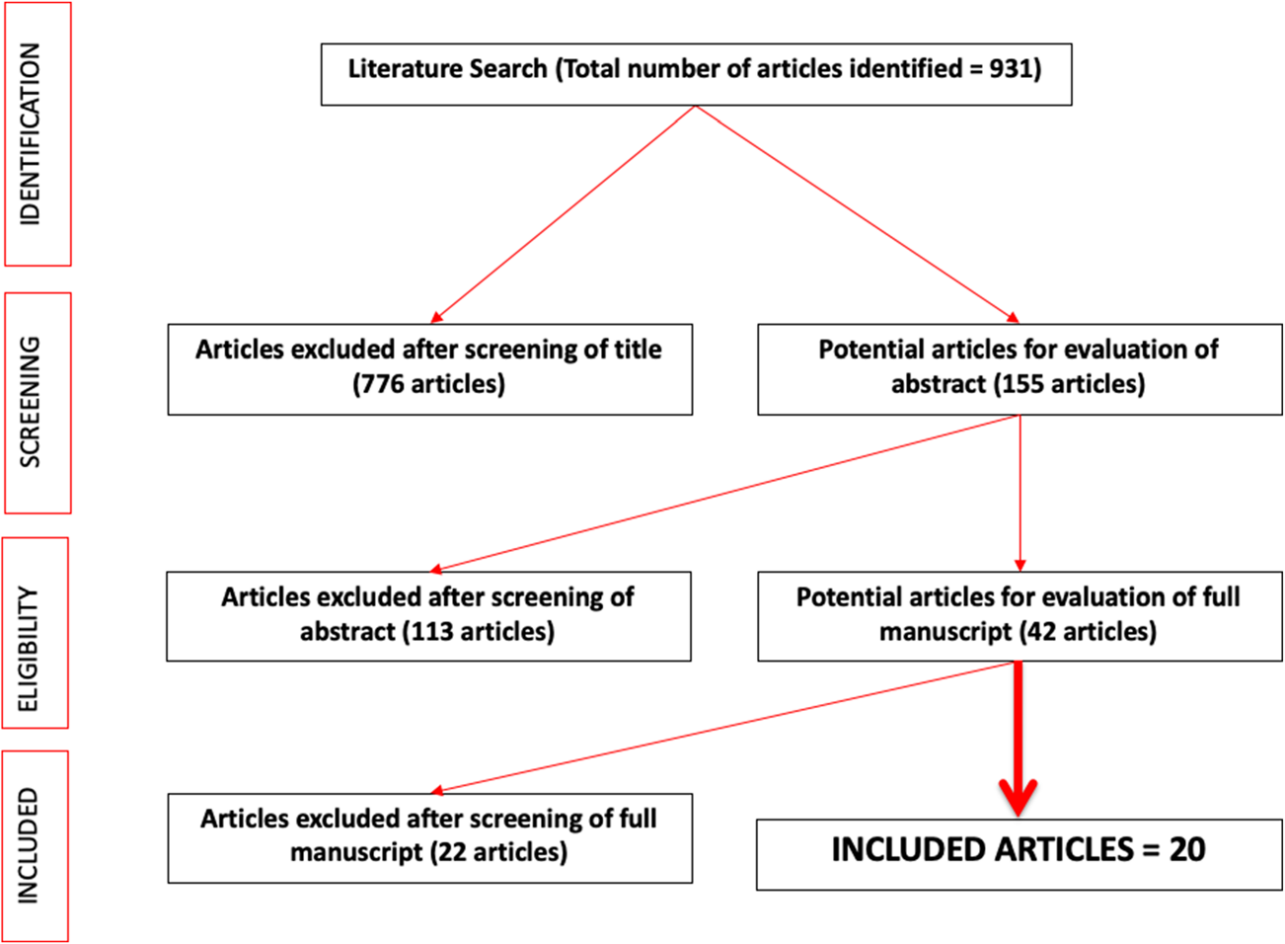
PRISMA flowchart of the included studies

Our search produced 931 articles of which 155 abstracts were reviewed and 20 articles (198 patients) met our inclusion criteria and were included in our final review (Fig. 1) [3–21, 25–27]. The removal of eroded sling/mesh was done by laser incision in nine studies, endoscopic approach using transurethral resection (TUR) or cutting it with scissors in seven studies and a mixture of the above two techniques in the remaining four studies (Table 1). In the reported studies, 108 (55%) patients were treated exclusively with laser excision and 90 (45%) were managed by intravesical resection of the mesh with electrode loop via a resectoscope, laparoscopic scissors or forceps.

**Table 1:** Summary of demographics and presentation

Reference/ year	Journal	Level of evidence	Number of patients	Age in years (median, range)	Initial procedure (number of patients)	Presenting symptoms	Time to presentation after surgery (months) (median, range)
Wang et al. 2018	Lower Urinary Tract Symptoms	4a	12	54, 40–66	TVT (2), TOT (4), mini-invasive (4), Solyx (1), unspecified (1)	SUI; UI; dyspareunia; voiding LUTS; mesh-associated stones	47, 10–161
Chan et al. 2016	Journal of Endourology	4a	18	58, 50–60	TVT (12); Burch/MMK (5); Bladder neck sling (1)	Voiding LUTS; pelvic pain; SUI; rUTIs; mesh-associated stones	84, 24–132
Lee et al. 2017	Female Pelvic Medicine & Reconstructive Surgery	4a	22	55.4, 43–68	TVT (2); TOT (16); transvaginal mesh repair for pelvic organ prolapse (4)	Voiding LUTS; haematuria; recurrent cystitis; dysuria; mesh-associated stones	63.6, 9–113
Ogle et al. 2017	Int Urogynecol J, Wiley	4a	10	58, 54–66	TVT (4), other urethral slings (5), MMK (1)	Voiding LUTS; pelvic pain; haematuria; rUTI	12, 1–72
Campobasso et al. 2014	Int Urol Nephrol	4a	3	62, 57–65	TVT	Voiding LUTS; haematuria; dysuria	Range: 60–120
Davis et al. 2012	The Journal of Urology	4a	12	55, 47–73	TVT (10), Burch (1), vesicopexy (1)	Voiding LUTS; SUI; rUTIs; haematuria	19, 3–84
Jo et al. 2011	Korean Journal of Urology	4a	23	51, 40–67	TVT (8), TOT (11), mini-sling (1), unknown (3)	Voiding LUTS	31, 2–247
Doumouchtsis et al. 2010	BJUI International	4a	6	64, 49–79	TVT (5), TOT (1) Burch (1)	Voiding LUTS; rUTIs	84, 12–156
Sakalis et al. 2011	Int Urogynecol J, Wiley	4a	7	58, 42–72	TVT	Voiding LUTS; dysuria; haematuria; rUTI; dyspareunia	25, 8–48
Frenkl et al. 2008	Neurourology and Urodynamics	4a	11	51, 41–84	Urethral sling, sacrocolpopexy, colposuspension	Voiding LUTS; dyspareunia; haematuria; SUI	35, 3–185
Giiri et al. 2005	The Journal of Urology	4a	3	50, 47–57	TVT, Burch, vesicopexy	Voiding LUTS; haematuria; rUTIs	Range: 1–12
Hodroff et al. 2004	The Journal of Urology	4a	3	Unknown	TVT	SUI, haematuria	Range: 1–6
Goujon et al. 2018	J Gynecol Obstet Hum Reprod	4a	13	58, 43–76	TOT (8); TVT (5)	Voiding LUTS; UI; rUTI; dysuria; pelvic pain; persistent SUI; haematuria	23, 0–127
Wijffels et al. 2008	Int Urogynecol J, Wiley	4a	3	52, 44–59	TVT	UI; SUI; rUTIs	Range: 6–14
Oh and Ryu 2009	Journal of Endourology	4a	14	48, 41–64	TVT (11), TOT (3)	Voiding LUTS; UI	5, 0–36
Velemir et al. 2008	Int Urogynecol J, Wiley	4a	4	54, 39–66	TVT (1), TOT (1), retropubic tape inserted trans-abdominally (2)	Voiding LUTS; rUTI	34, 2–180
Huwyler et al. 2008	BJUI International	4a	5	68, 63–74	TVT	Dyspareunia; rUTI; UI	Range: 1–18
Baracat et al. 2005	CLINICS	4a	11	Unknown	TVT	Voiding LUTS; rUTI	Unknown
Foley et al. 2010	BJUI International	4a	9	60, 38–75	TVT (8), TOT (1)	Voiding LUTS, rUTI	8, 2–18
Castroviejo-Royo et al. 2015	Journal of Endourology	4a	9	61, 49–70	TOT (3), mini-sling (6)	Voiding LUTS	13,1–79

*TVT* tension-free vaginal tape, *TOT* transobturator tape, *UI* urge incontinence, *SUI* stress urinary incontinence, *LUTS* lower urinary tract symptoms, *rUTI* recurrent urinary tract infections

### Demographics and clinical presentation

All of the included articles were retrospective case series and reported on 3–23 patients each. The mean age across studies varied from 48 to 68 years, and 149 (75%) had tension-free vaginal tapes (TVT) or transobturator tapes (TOT) as their initial procedure. The journal, year of publication, level of evidence and time to presentation after their original surgery are reported in Table 1.

Voiding lower urinary symptoms (LUTS) was the most commonly reported symptom (17 studies). Other symptoms included recurrent UTIs (11 studies), haematuria (9 studies), SUI (6 studies), urge incontinence (UI) (5 studies), dyspareunia (7 studies), dysuria (4 studies) and pelvic pain (3 studies). Mesh-associated stones were a common finding across the literature in patients with urinary mesh erosion. The median time to presentation after their initial surgery was 34.5 months (range: 1–247 months).

### Site of mesh erosion

The site of mesh erosion was the bladder in 134 patients (68%), of which 12 (6%) were in the bladder neck. Urethral mesh erosion was seen in 63 patients (32%) across all studies.

### Outcomes with laser incision

One hundred eight (55%) patients were treated exclusively with laser incision (Table 2). The initial and final success rate with this technique was 67% (72 patients) and 92% (99 patients) respectively. Of these patients, 27 needed further laser procedures, with 18 (17%) needing one additional procedure, 6 (5.6%) needing two additional procedures and 3 (2.8%) needing multiple (> 2) procedures. Of the remaining nine patients, eight (7%) needed vaginal surgery to remove a mesh remnant and one needed open cystotomy to remove the eroded mesh.

**Table 2:** Summary of procedures, outcomes and complications

Reference/ year	Number of patients	Location of mesh erosion	Type of endoscopic procedure	Follow-up duration (months)	Complications (number of patients)	Management of complications	Recurrence of mesh erosion (number of patients)	Repeat operations (number of patients)	Initial success rate	Final success rate
Wang et al. 2018	12	U	Holmium laser	14–70	None reported	None	6	Multiple repeat procedures (1); vaginal surgery (5)	50%	58%
Chan et al. 2016	18	B (6) BN (4) U (8)	Holmium laser	12–24	Worsening SUI (7), new SUI (1), post-op UTI (2)	None	4	1 Repeat procedure(3); 2 repeat procedures (1)	78%	100%
Lee et al. 2017	22	B (16) U (6)	Holmium laser	23.2	SUI (5)	None	6	1 repeat procedure (5); 2 repeat procedures (1)	73%	100%
Ogle et al. 2017	10	B (6) U (4)	Holmium laser	27	SUI (3)	None	3	1 Needed further procedure for SUI	60%	100%
Campobasso et al. 2014	3	B	Holmium laser ± endoscopic forceps	15	Unknown	None	1	1 Recurrent mesh treated with repeat procedure	67%	100%
Davis et al. 2012	12	B (10) U (2)	Holmium laser (10), thulium laser (2)	6–134	SUI (2)	None	5	1 Repeat procedure (4); open cystostomy (1)	58%	92%
Jo et al. 2011	23	B (20) U (3)	TUR with electrode loop (16); Holmium laser(7)	2	Vesicovaginal fistula following TUR with electrode (2); SUI (1)	Fistula repair	6	TURE: 1 Further procedure for mesh removal (1) TURH: transvaginal removal (3); repeat procedure (2)	74%	87%
Doumouchtsis et al. 2010	6	B (2) U(4)	Holmium laser	12–36	SUI (1), voiding difficulty(1)	None	3	1 Needed partial cystectomy, repeat procedure (2)	50%	83%
Sakalis et al. 2011	7	B	Holmium laser	12	None reported	None	0	Nil	100%	100%
Frenkl et al. 2008	11	B (9) BN (7) U (6)	Holmium laser (4), scissors (4), endoscopic TUR (2)	Unknown	Unknown	None	2	Repeat procedure (2)	82%	100%
Giiri et al. 2005	3	B	Holmium laser	3–12	SUI (1)	None	0	Nil	100%	100%
Hodroff et al. 2004	3	B (2) BN (1)	Holmium laser	1	SUI (2)	None	0	Nil	100%	100%
Goujon et al. 2018	13	B (10) U (3)	Cystoscopy + suprapubic percutaneous access + laparoscopic scissors/holmium laser	6	Intra-peritoneal bladder perforation (1); retroperitoneal bladder perforation (1); vesicovaginal fistula (1)	Conversion to laparoscopy for diagnosed perforation (2); fistula repair (1)	6	2 Repeat procedures (4); 5 procedures (1); 6 procedures (1)	54%	100%
Wijffels et al. 2008	3	U	Endoscopic scissors	Unknown	SUI (2)	None	1	Repeat procedure (1)	67%	100%
Oh and Ryu 2009	14	B	Endoscopic TUR	3–12 Cystoscopy; 5–40 (clinical)	Vesicovaginal fistula (1); full-thickness perforation and haematoma (1); mild SUI (1); UUI (1)	Fistula repair; Surgical drainage of haematoma	1	Repeat procedure (1)	93%	100%
Velemir et al. 2008	4	U	Endoscopic TUR	6–24	SUI (3)	None	2	Repeat procedures (2)	50%	100%
Huwyler et al. 2008	5	B	TUR	3–23	SUI (1)	None	0	Nil	100%	100%
Baracat et al. 2005	11	B (6) U (5)	Bladder erosion-lap-assisted endoscopic excision; urethral erosion-endoscopic excision	6	SUI (3)	None	1	Repeat procedure (1)	91%	100%
Foley et al. 2010	9	B (6) U and B (3)	Endoscopic TUR or direct excision with nasal speculum	Unknown	SUI (9)	None	4	Repeat procedure (2); open surgery (2)	56%	78%
Castroviejo-Royo et al. 2015	9	B	TUR with electrode loop	38	None reported	None	0	Nil	100%	100%

*B* bladder, *BN* bladder neck, *U* urethra, *SUI* stress urinary incontinence, *UUI* urge urinary incontinence, *TUR* transurethral resection

Post-operative complications were seen in 26 (24%) patients. These included SUI in 23 (21%), urinary tract infection in 2 (1.9%) and voiding difficulty in 1 patient. There were no other reported major complications such as fistula formation or intra-operative perforation.

### Outcomes with endoscopic procedure using resectoscope/electrode loop or laparoscopic scissors/forceps

Ninety (45%) patients were managed using TUR with an electrode loop or laparoscopic scissors/forceps. The initial and final success rates with this technique were 80% (72 patients) and 98% (88 patients) respectively. Of these patients, 16 needed further endoscopic procedures, with 10 (11%) needing one additional procedure, 4 (4%) needing two additional procedures and 2 (2%) needing multiple (> 2) procedures. The remaining two patients needed open surgery to remove the eroded mesh.

Post-operative complications were seen in 25 (28%) patients. These included SUI in 19 (21%), vesicovaginal fistula in 3 (3%), intra-peritoneal bladder perforation needing a further procedure in 2 (2%) and retroperitoneal bladder perforation in 1 patient.

## Discussion

### Meaning of the study

While there is increasing concern about the growing numbers of mesh or sling erosions, all types of surgical intervention carry a risk of morbidity. Bearing in mind the limitations of our review, which is based on retrospective case series, endourological techniques show excellent success rates of > 90% with endoscopic techniques using laser excision or endoscopic resection, although 20–25% patients may need more than one endoscopic procedure to achieve this. Considering the lack of an open comparator group and reporting bias, the real success rate could be much lower. While the complication rates seem to be 24–28%, one would argue that the majority of these were SUI, which is expected as an outcome of mesh removal irrespective of whether it is done endoscopically or as an open approach, and this may not really count as a complication but probably as an expected outcome of the mesh removal procedure.

### Outcomes with practical considerations related to mesh erosion

Polypropylene meshes play a huge role in the minimally invasive repair of pelvic organ prolapse (POP) and SUI [28]. However, this also presents complications of mesh misplacement, migration or erosion [4]. FB erosion following urinary incontinence surgery can be a late complication and may be associated with the material used, operating surgeon’s expertise and technical difficulties at the time of initial surgery [4, 13]. Unrecognized bladder perforation during trocar insertion can also cause mesh erosion and may present as a late complication [3].

Often presenting with voiding LUTS, recurrent UTIs and haematuria, other symptoms identified in our review included SUI, urge incontinence (UI), dysuria and dyspareunia. The median time of presentation after surgery was 34 months, and a low index of suspicion is necessary in these patients with a previous history of incontinence-related procedures. This delay in presentation can be due to vague non-specific symptoms and a lack of standardized follow-up [29]. A high index of suspicion is often needed, and a detailed evaluation is necessary often with a cystoscopy to examine the LUT, which can help to confirm the diagnosis. Mesh-associated stones are not uncommon and the presence of stones should raise a strong suspicion of mesh erosion.

Our review of the literature, which is the largest to date, shows that endoscopic management is a feasible and effective option in managing FB erosion. Holmium laser was associated with an overall success rate of 92% and endoscopic management with resectoscope/laparoscopic scissors had a success rate of 98%. The most common complication was SUI in both groups; however it was largely unclear whether this was de novo or a pre-existing complaint. SUI following removal of mesh surgery can be due to a failed incontinence procedure but equally mesh erosion can damage the urethra and sphincter resulting in scarring. The management of this can be complicated and may need reconstructive surgery with urethroplasty to help improve functional outcome [4]. Endoscopic management with resectoscope and electrode loop and the use of laparoscopic scissors was associated with vesicovaginal fistula in three patients and bladder perforation in two patients. It is worth noting that the laser approach allowed for decreased post-operative morbidity and complications, decreased inpatient hospital stay and good functional outcomes [7]. However, the outcomes of the two approaches may not be directly comparable because of different populations, variation in techniques and different surgical expertise.

### Implications for clinical practice

The duration of follow-up varied across all studies and ranged from 1 to 134 months. We suspect it is important for all patients to have long-term outpatient-based follow-up and perhaps have a vaginal speculum examination along with a flexible cystoscopy 6–12 months post-operatively to pick up any early signs of mesh erosion [30]. This could be repeated if there are any obvious symptoms or a clinical suspicion of erosion. Presence of bladder or urethral stones especially if adherent to the musoca suggests an erosion. The patients should also be counselled to return for a review if they develop persistent or worsening urinary symptoms. Patients at high risk of potential mesh-related complications seem to be those who have a combined mesh for POP repair and SUI mesh sling [30]. In a study of 41,604 women in the US who underwent transvaginal mesh repair for POP with or without concurrent sling for SUI, transvaginal POP repair without mesh but with concurrent sling or a sling for SUI only and a follow-up period of 1-year, the risk of erosion was 2.7% and the risk of repeated surgery with concomitant erosion was 2.1% in the POP repair with mesh plus sling group [30]. The SUI sling group on its own had an erosion rate of 1.5% and a risk of repeated surgery with concomitant erosion of 1.6%. Similarly, concomitant hysterectomy and hypertension were also associated with mesh erosions [31]. In high-risk patients the index of FB erosion and early counselling and appropriate management should be made available.

### Strengths, limitations and areas of future research

In this review we adhere to the methodological approach of the Cochrane guidelines and PRISMA checklist. It summarizes the role of endoscopic management of mesh erosions including the use of both laser and transurethral resection and gives an overview of the success rate and complications with this technique, although all papers included in our review were retrospective case series and hence prone to bias. Furthermore, there was a lot of heterogeneity in the reported studies making it challenging to do a formal meta-analysis. There are some limitations to our systematic review. Individual studies did not have a standardized management or follow-up, and this was left to the individual surgeon and centre based on their expertise and patient presentation. Given that data collection and reporting were not standardized either, it was difficult to compare or combine outcomes.

A recent study looked at the reporting outcome measures in trials on synthetic mesh procedures for POP and concluded that urgent action is needed to improve the quality of research in this field [32]. Of the 71 randomized trials, 24 different types of mesh were identified. These trials reported on 110 different outcomes and 60 outcome measures. Clinically important measures such as erosion, pain and dyspareunia were reported in 40, 29 and 25 trials respectively. They recommend developing and implementing a minimum standardized data set, which forms the core outcomes of this procedure. Another systematic review on the type of synthetic material used found that polyester sling material caused the highest rates of vaginal erosion [33]. The US Food and Drug Administration (FDA) has ordered all manufacturers of surgical mesh intended for transvaginal repair of prolapse (cystocoele) to stop selling and distributing the product [34]. Given the scale of mesh erosions and the public outcry associated with it, perhaps a national registry of all sling or mesh procedures should be the way ahead, where every case is registered and the core outcome measures including the type of material used, surgical technique, complications and follow-up are mandatory. This should be protocol based and have outcome measures which are standardized and hence comparable.

## Conclusions

Endoscopic management of FB erosion is an effective minimally invasive technique with good outcomes and minimal morbidity, which uro-gynaecologists or endourologists might be able to offer as a treatment option. Management with the use of holmium laser is gaining momentum and could be attempted before open surgical removal. There is a need for comparative data between open surgical excision and endoscopic excision to help better describe the patient’s most likely to benefit from the endoscopic technique.
